# Health promotion and the agenda for sustainable development, WHO Region of the Americas

**DOI:** 10.2471/BLT.17.204404

**Published:** 2018-07-27

**Authors:** Kira Fortune, Francisco Becerra-Posada, Paulo Buss, Luiz Augusto C Galvão, Alfonso Contreras, Matthew Murphy, Caitlin Rogger, Gabriela E Keahon, Andres de Francisco

**Affiliations:** a Family, Health Promotion, and Life Course, Pan American Health Organization, 525 23rd Street, Washington, DC 20037, United States of America (USA).; b Assistant Director Office, Pan American Health Organization, Washington, USA.; c Centre for International Relations in Health, Fundação Oswaldo Cruz, Rio de Janeiro, Brazil.; d Independent consultant, Washington, USA.; e Department of Internal Medicine, Brown University, Rhode Island, USA.

## Abstract

The approaches and tools of health promotion can be useful for civil society groups, local and national governments and multilateral organizations that are working to operationalize the 2030 agenda for sustainable development. Health promotion and sustainable development share several core priorities, such as equity, intersectoral approaches and sustainability, that help maximize their impact across traditional sectoral boundaries. In the Region of the Americas, each of these priorities has strong resonance because of prominent and long-standing health inequities that are proving resistant to interventions driven solely by the health sector. We describe several cases from the World Health Organization’s (WHO) Region of the Americas in which the approaches and tools of health promotion, with a focus on cities, healthy settings and multisectoral collaboration, have been used to put the agenda into practice. We highlight areas where such approaches and tools can be applied effectively and provide evidence of the transformative potential of health promotion in efforts to achieve the sustainable development goals.

## Résumé

Les approches et outils de promotion de la santé peuvent être utiles aux groupes de la société civile, aux gouvernements locaux et nationaux et aux organismes multilatéraux qui s'efforcent de mettre en œuvre le Programme de développement durable à l'horizon 2030. La promotion de la santé et le développement durable ont plusieurs priorités centrales en commun, telles que l'équité, la collaboration intersectorielle et la durabilité, qui contribuent à optimiser leur impact au-delà des limites sectorielles habituelles. Chacune de ces priorités a une forte résonance dans la Région des Amériques, où les importantes inégalités en termes de santé durent depuis longtemps et semblent résister aux interventions menées à la seule initiative du secteur de la santé. Dans cet article, nous décrivons divers exemples provenant de la Région des Amériques de l'Organisation mondiale de la Santé (OMS), où plusieurs approches et outils de promotion de la santé (ciblant principalement les populations urbaines, la création d'environnements-santé et une collaboration multisectorielle) sont actuellement employés pour mettre en œuvre le Programme de développement durable. Nous mettons l'accent sur les domaines dans lesquels ces approches et outils peuvent être efficacement appliqués et nous proposons des données probantes sur le potentiel de transformation de la promotion de la santé dans les efforts menés pour atteindre les objectifs de développement durable.

## Resumen

Los enfoques y las herramientas para el fomento de la salud son útiles para los grupos de la sociedad civil, los gobiernos locales y nacionales y las organizaciones multilaterales que trabajan para poner en marcha el programa de desarrollo sostenible de 2030. El fomento de la salud y el desarrollo sostenible comparten varias prioridades básicas, como la equidad, los enfoques intersectoriales y la sostenibilidad, que ayudan a maximizar el impacto dentro de los límites sectoriales tradicionales. En la Región de las Américas, cada una de estas prioridades tiene una fuerte repercusión debido a las inequidades sanitarias prominentes y prolongadas que resisten a las intervenciones impulsadas únicamente por el sector de la salud. Se describen varios casos de la Región de las Américas de la Organización Mundial de la Salud (OMS) en los que los enfoques y las herramientas para el fomento de la salud, centrado en ciudades, entornos saludables y colaboración multisectorial, se han utilizado para poner en práctica el programa. Se destacan las áreas donde tales enfoques y herramientas se pueden aplicar de manera efectiva y ofrecer pruebas del potencial transformador del fomento de la salud en los esfuerzos por alcanzar los objetivos de desarrollo sostenible.

## ملخص

يمكن أن تكون الأساليب والأدوات المستخدمة للنهوض بالصحة مفيدة لجماعات المجتمع المدني والحكومات المحلية والوطنية والمنظمات متعددة الأطراف والتي تعمل لوضع جدول أعمال التنمية المستدامة لعام 2030 قيد التنفيذ. يشترك النهوض بالصحة والتنمية المستدامة في العديد من الأولويات الأساسية كالمساواة والأساليب المشتركة بين القطاعات والاستدامة، والتي تساعد في تعظيم أثرها عبر الحدود القطاعية التقليدية. وفي منطقة الأمريكتين يكون لكل واحدة من هذه الأولويات صدى قوي بسبب الحالات البارزة وطويلة الأمد لعدم المساواة في المجال الصحي والتي تبرهن على مقاومة التدخلات التي يقودها القطاع الصحي بمفرده. ونحن نصف حالات عديدة في منطقة الأمريكتين والتي يشملها نطاق عمل منظمة الصحة العالمية حيث تم استخدام الأساليب والأدوات المستخدمة للنهوض بالصحة، مع التركيز على المدن والأوضاع الصحية والتعاون المتعدد القطاعات، لوضع جدول الأعمال موضع التنفيذ. ونقوم بتسليط الضوء على مناطق يمكن فيها تطبيق هذه الوسائل والأدوات بفاعلية ونقدم الدليل على إمكانية إحداث تغيير للنهوض بالصحة في إطار الجهود الرامية لتحقيق أهداف التنمية المستدامة.

## 摘要

促进卫生的方法和工具对于正在努力落实 2030 年可持续发展议程的民间社会团体、地方和国家政府以及多边组织均有用。促进卫生和可持续发展具有几个核心重点，例如平等、跨部门的方法和可持续发展，这有助于最大限度地发挥他们的影响力，突破传统部门界限。在美洲地区，由于突出和长期存在的卫生不平等现象，每一个重点都有很强的相关性，这些不平等现象证明卫生部门完全难以推动干预措施。我们描述了世界卫生组织美洲地区的几个案例，现已将其中以城市、卫生状况和跨部门协作为重点的促进卫生方法和工具用来实施议程。我们强调可以有效应用这些方法和工具的领域并提供证明在实现可持续发展目标方面努力促进卫生的变革潜力。

## Резюме

Подходы и инструменты, используемые для пропаганды здорового образа жизни, могут принести пользу представителям гражданского общества, государственным учреждениям и органам местного самоуправления, а также многосторонним организациям, которые работают над реализацией повестки дня в области устойчивого развития на период до 2030 года. Пропаганда здорового образа жизни и устойчивое развитие имеют несколько основных приоритетов, таких как равенство и справедливость, межсекторальные подходы и устойчивость, которые помогают максимально усилить их воздействие и преодолевать традиционные границы между секторами. В американском регионе каждый из этих приоритетов имеет сильный резонанс из-за значительного и долговременного неравенства в области здравоохранения, которое оказалось устойчивым к вмешательствам, осуществляемым исключительно в секторе здравоохранения. Авторы описывают несколько примеров из стран американского региона Всемирной организации здравоохранения (ВОЗ), в которых для реализации повестки дня на практике были использованы подходы и инструменты пропаганды здорового образа жизни, при этом особое внимание уделялось городам, здоровому окружению и межсекторальному сотрудничеству. Были выделены области, где можно эффективно применять такие подходы и инструменты, и приведены доказательства того, что пропаганда здорового образа жизни может помочь увидеть изменения в усилиях по достижению целей в области устойчивого развития.

## Introduction

*Transforming our world: the 2030 agenda for sustainable development* ^1^ set the scene for innovative approaches to tackling inequities in health. Public authorities and civil society are encouraged to adapt the aspirational and ambitious, equity-focused vision of the agenda and its 17 sustainable development goals (SDGs)^2^ to local and national health priorities. Health promotion, that is, the process of enabling people to increase control over, and to improve, their health,^3^ has a potentially transformative role to play. As an approach, it aims to alter the economic, environmental, institutional and social contexts in which decisions relating to health and well-being are made, while sharing the SDGs’ focus on equity.

The scale of the transformation required to achieve all of the SDGs is considerable. Historically, in general, sector-specific commitments have driven the actions of the key players in international development. The millennium development goals (MDGs)^4^ led to great gains in terms of the mean levels of national health-related performance indicators, most of them disease-specific. However, the MDGs also reinforced the entrenched sector-specific modalities of working that the SDG agenda seeks to change. Under the agenda, the MDGs’ exclusive focus on low- and middle income countries has evolved into a systemic, whole-society approach that seeks to reduce inequality within and among countries and establish greater opportunities for comprehensive change. In response, the World Health Organization (WHO) has established six lines of action (Box 1) and a series of explicitly multisectoral tools to approach the breadth of the health-related SDGs.^5^

Box 1 **World Health Organization’s six lines of action to promote health in the agenda for sustainable development, 2017^5^**

1. Intersectoral action by multiple stakeholders

2. Health systems strengthening for universal health coverage

3. Respect for equity and human rights

4. Sustainable financing

5. Scientific research and innovation

6. Monitoring and evaluation

Health promotion has a fundamental role to play in realizing the entire agenda. In contrast to the MDGs, the agenda highlights health as a component of all the SDGs and a critical element of the process of developing an equitable and sustainable future. Compared with the MDGs, SDG 3, which aims to “ensure healthy lives and promote well-being for all at all ages”, applies a much more expansive view of health. The direct or indirect links of health to all 17 SDGs highlight both the complex role and the importance of health promotion in achieving equity, empowering communities and people and protecting human rights.

It has been argued that the agenda places too much focus on relatively narrow measures of economic performance, e.g. economic productivity, gross domestic products and job creation, and too little focus on sustainable environmental and social measures.^6^ Such focus is even greater for SDG 8, which aim is to “promote sustained, inclusive and sustainable economic growth, full and productive employment and decent work for all”. Any bias towards economics may invite conceptual conflicts with the theory and practice of health promotion, as well as with SDG 3’s explicit emphasis on well-being.^6^ Many of the targets outlined in the SDGs focus on the prevention of death and illness rather than on the promotion of overall health and well-being, and the generation of environments that have health benefits. The purpose of this article is not to argue that the various approaches towards achievement of the 17 SDGs are mutually exclusive or uniformly synergistic, but rather to highlight the opportunities to achieve the overarching aims of the SDGs by use of the tools of health promotion.

The vision for the agenda was outlined in a document that the United Nations published in 2012: *Realizing the future we want for all*.^7^ In placing the three fundamental principles equality, human rights and sustainable development at the core of the agenda, this document shared some of the key goals and values of the health-promotion movement. In November 2016, the Ninth Global Conference on Health Promotion highlighted the closely intertwined priorities of the health-promotion and sustainable-development movements, particularly the reduction of the inequity that hampers attempts to achieve several wide development aims.^8^ The reduction of inequity has particular resonance in the WHO Region of the Americas, which remains one of the most inequitable regions. In Latin America and the Caribbean in 2014, about 29% of the population lived below the poverty line and the poorest 40% of the population received less than 15% of the total income.^9^

## Tackling health inequities

Measurement of progress towards the MDGs was mostly reported as aggregated means that failed to illustrate the contributions that various subgroups of the population made towards a specific outcome. As the MDGs did not effectively address the differentials in health status associated with educational, ethnic, gender, socioeconomic and other differences, they often allowed health inequities to persist. However, they also led to much success. Within the Region of the Americas, for example, many countries reached or surpassed the MDGs relating to access to education, improved water and sanitation.^10^ Among other progress, the region met the target of reducing, by two thirds, mortality among children younger than 5 years. Between 2000 and 2015, such mortality fell from 54 to 17 deaths per 1000 births and the percentage of the region’s children who were underweight decreased from 7.3% to 2.3%.^11^

The Region of the Americas faces several challenges. While rapid economic development has opened the window to many health gains, socioeconomic inequalities combined with other important determinants of health, e.g. education, ethnicity and gender, are associated with considerable gaps in health outcomes and unacceptable health inequities. For example, life expectancy at birth in Canada is nearly 20 years longer than that in Haiti and infant mortality rates in Canada and Cuba are about a tenth of those in the Plurinational State of Bolivia and Haiti.^11^ In 2015, when the MDGs should have been achieved, there were still between-country differences in the numbers of maternal deaths per 100 000 live births. In that year, such maternal mortality rates for many of the region’s Member States were either far below or far above the mean rate for the region.^12^ Furthermore, while the region met the MDG target for reducing the proportion of people living in urban slums, there was a concurrent increase in the total number of people living in such slums.^10^

In addition to the need to address health inequities, the gains under the MDGs also call for changes in priority health areas. For example, during attempts to achieve the MDGs, the Region of the Americas saw a substantial increase in the proportional contribution that neonatal mortality made to mortality among children younger than 5 years. This observation indicates the need for a greater focus on neonatal mortality and its associated risk factors. Although the development of enhanced immunization programmes has led to substantial increases in coverage of three doses of diphtheria–pertussis–tetanus vaccine, about one million children are still unvaccinated in the region, many of them difficult to reach and living in situations of vulnerability.^13^ Communities in the region, and the health systems that serve them, also face growing and costly threats to health in the form of increases in the incidence of cancer, cardiovascular and chronic respiratory diseases, diabetes and other noncommunicable diseases. Together, such diseases accounted for 79% of all deaths in the region in 2012.^14^

## Promoting health via the SDGs

Over the last few decades, several new approaches to health promotion have been developed. Most have involved the integration of multilevel interventions. There has been an emphasis on enabling healthy environments, reorienting health services and promoting well-being and healthy choices via community involvement, public policies and the strengthening of individual capacity to control the determinants of health.^15^^,^^16^ Given their broad scope, health-promotion initiatives can support diverse benefits for both health and sustainable development.

The Ninth Global Conference on Health Promotion highlighted various approaches to health promotion, based on public policy, that were predicted to facilitate achievement of the SDGs. These approaches included action across sectors, the development of so-called healthy cities and social mobilization.^8^ In a reaffirmation of the need for the healthy public policy described in the Ottawa Charter decades previously, the conference’s published Shanghai Declaration expressed the need for the global community to implement integrated and strategic approaches that harness synergies across multiple sectors and deliver collaborative gains.^17^ Recognizing the increasingly salient role played by the commercial determinants of health, the declaration’s signatories agreed to emphasize good governance for health via the strengthening of legislation on, and the regulation and taxation of, unhealthy commodities.

The development of healthy settings represents a currently widespread strategy for putting health promotion into practice at the country level with important ties to universal health coverage and access to key health and social interventions.^18^^–^^20^ In particular, cities, communities and municipalities are emerging as key contexts for achieving the SDGs via the use of both familiar and new health-promotion tools. The settings approach has, however, experienced various challenges from which the health community should learn. The achievement of sustainable change via multisectoral action can be difficult to demonstrate, or even implement in a short time frame. In any short-term assessment, process indicators may be appropriate measures of success. Given their widespread acclaim, the evidence base for the effectiveness of healthy-settings approaches remains surprisingly limited.^21^ To generate more relevant evidence, which is clearly linked to policy and practice, governments at all levels must be actively engaged in long-term monitoring and evaluation.^22^^,^^23^

The Shanghai Declaration also recognized the need for more promotion of health literacy. By strengthening health literacy at local and national levels, it should be possible to increase each person’s control over their own health and their capacity to engage with the broader determinants of health.^24^

## Approach to health equity

A growing number of initiatives, spearheaded by national and local policy-makers, highlight how the approaches we have discussed can be translated into action in the Region of the Americas. Mexico’s National Agreement for Healthy Food represents a notable example of successful intersectoral action in the region.^25^ This agreement was launched in 2010, in response to a growing epidemic of obesity in Mexico, particularly among children and adolescents. After recognizing the epidemic as a costly and unsustainable threat to health, the Mexican government mobilized the heads of 15 different government agencies under the stewardship of the health ministry. The aim was to develop intersectoral solutions to the crisis, using the guiding principles of equity, gender, interculturalism and social inclusion. The result has been the initiation of more than 100 activities across different sectors, with active participation from the private sector.^25^ The activities include the provision of healthy foods in schools, the restriction of sales of processed foods at schools, the updating of regulations on processed foods and the rehabilitation of public spaces to promote physical activity.

The movement for healthy cities, communities and municipalities has also attracted considerable enthusiasm. The Shanghai Consensus on healthy cities, which was adopted at the Ninth Global Conference on Health Promotion in 2016, highlights the strong links between sustainable urban development and health.^26^ In 2018, the Copenhagen Consensus, on healthier and happier cities for all, detailed several key opportunities for achieving progress across a range of needs, from pressing health challenges such as climate change and noncommunicable diseases to the broader promotion of well-being.^27^

There have also been encouraging initiatives at the local level. For example, the Colombian city of Medellín underwent a process of citizen and urban transformation in 2004 and has since been increasingly recognized as a hub for civic engagement, equity, social and technological innovation and sustainability.^28^ The city has implemented numerous programmes to promote sustainability by addressing the inequities in access to education, housing and infrastructure and employment opportunities. The result has been a reduction in crime, improvements in economic growth, increased investment in public infrastructure and, apparently, improvements in the quality of life of the city’s residents. Each year, Medellín’s metro system transports over 160 million passengers and uses clean and efficient systems to save over 178 000 tonnes of carbon-dioxide emissions, while also reducing road traffic accidents and traffic in the city.^29^ Medellín is also one of the only cities in Colombia to develop and implement a science, technology and innovation plan.^28^ The city has structured its interventions using operational models that foster intersectoral actions across governments and the development of partnerships with academia and the private sector.^30^

In 2013, Chile established a healthy cities, communities and municipalities strategy, with the goal of promoting intersectoral collaborations to address pressing local health needs. Since then, several national ministries have coordinated with over 300 municipalities to implement programmes that address inequities in access to healthy food and public health services.^31^ Two key aspects of the strategy were the development of national intersectoral agreements and political commitments, with the support of mayors, and the enhancement of the coordination between the local and national actors. Constant dialogue across all levels of government has helped ensure synergy between all of the strategy-related actions, decisions and policies.

The observations made in Chile and Colombia highlight some of the important contributions that cities and municipalities are making towards health and the SDGs. There is a powerful link between the health-related SDG 3 and SDG 11, which promotes sustainable cities and communities. The substantive leadership role played by mayors and municipal leaders, both individually and in formal and informal networks, appears to be critical to the success of healthy-city initiatives. Mayors can play a defining role in creating healthy urban environments through determined political action and are also often able to lead in the delivery of SDG-related activities.^26^

## Discussion

The ultimate aims of the agenda are to safeguard the planet and improve people’s lives. The agenda’s goals, indicators and targets should reflect the efforts that each country, local community and individual are making to improve global health and not, simply, the ability of a single sector to achieve a few specific outcomes.^32^

There are several obstacles facing the achievement of the SDGs. Given the substantial financial backing and resources required for implementing SDG-related strategies and policies, sustainable development requires action from a broad range of investors.^33^ Governments need to mobilize sustained financial support from the private sector and from domestic sources such as taxation.^34^ The development of integrated strategies that leverage funding for health through multisectoral collaboration, e.g. building family planning into strategies for financing adaptation to climate change, is gaining interest.^35^ Effective health promotion, which has been shown to reduce the long-term costs of health care in multiple settings, must become a central part of such strategies.^36^^–^^39^

Once each country decides on which SDGs and targets they will focus on, a core challenge will be the establishment of a strong framework to monitor progress in addressing those goals and targets, especially those related to health and inequalities. We need improvements in our understanding of successful interventions and in the identification of populations with the greatest needs. As a basic requirement for pursuing effective action under the SDGs, we also need to use existing data to build the evidence base for health-promotion actions and interventions that address determinants of health.

In many low- and middle-income countries, there are extreme shortages of relevant disaggregated data on educational level, ethnicity, socioeconomic status and other important health determinants.^40^ In response to these issues, the Pan American Health Organization (PAHO) has partnered with the Economic Commission for Latin America and the Caribbean to help build capacity for monitoring progress towards SDG 3 and other health-related indicators. PAHO will support the generation of disaggregated data by building capacity in data management, ensuring data quality and strengthening information systems for health and vital statistics.^40^ This and similar coordinated regional or global efforts will be crucial if countries are to have reliable and timely data for systematic follow-up and progress reviews. In turn, countries will need to build capacity for assessing the determinants of health and health inequalities, particularly those that relate to noncommunicable diseases and other priority areas. It will also be important to gather effective arguments for intersectoral work, drawing on the evidence from Medellín and other cities to inspire the scaling up of such work and the replication of the same approaches in additional settings.

## Conclusions

The health-promotion and sustainable-development movements can each be more successful if they build on their shared priorities, exploit the pool of mutually relevant knowledge and capitalize on growing global interest. Open and inclusive channels of communication need to be established among all of the groups that are devoting resources to accelerate the implementation of the agenda. Given the overlap of the goals, methods and priorities associated with the SDGs with those associated with health promotion, progress made on the SDGs has great potential to advance health promotion simultaneously and vice versa. Approaches that promote health are key to achieving the SDGs. Health equity comprises a core shared value that should, according to recent global commitments such as the Shanghai Consensus,^26^ guide policy-making in public health. The ability both to learn from scattered, practical examples of success and to document evidence of impact from diverse partners with different agendas and interests, will be key to maximizing the potential of health-promotion approaches in supporting the achievement of the SDGs.

Several challenges remain, particularly the dearth of frameworks for effective monitoring and evaluation and the dearth of evidence on the impacts of complex social actions on health determinants and systems. If the field of health promotion is to embrace its full potential role in responding to these challenges and achieving the SDGs, we need to build a solid evidence base and document and disseminate the relevant evidence that is already in existence. For health-promotion interventions, we need more data on the impact of multisectoral collaborations designed to support health and healthy settings and on the conditions that promote equity effectively.

By applying the tools and principles of these converging agendas, in systematic, targeted and measurable ways that reflect the mandates of recent global and regional commitments, it should be possible to make dramatic strides towards our shared vision of an equitable, healthy and sustainable future in which no one is left behind.
